# Periodontitis is associated with the increased levels of visfatin: a meta-analysis

**DOI:** 10.1186/s12903-023-03384-2

**Published:** 2023-10-26

**Authors:** Junfei Zhu, Suhan Zhang, Jing Shi, Ning ning, Ying Wei, Ye Zhang

**Affiliations:** 1 Stomatology Center, China Japan Friendship Hospital, Beijing, China; 2 Department of Dermatology, China Japan Friendship Hospital, Beijing, China; 3 The ward of stomatology center, China Japan friendship hospital, Beijing, China; 4 The Second Department of Proctology, China Japan Friendship Hospital, Beijing, China

**Keywords:** Visfatin, Periodontitis, Periodontal treatment, Gingival crevicular fluid, Meta-analysis

## Abstract

**Objective:**

Periodontitis is a common inflammatory disease associated with systemic factors. Visfatin is a pleiotropic adipokine that exerts metabolic and immune functions. Studies have shown visfatin played roles in the development of periodontitis. The present study aims to compare the levels of visfatin in body fluids including serum, saliva, and gingival crevicular fluid (GCF) between periodontitis patients and healthy individuals, and to elucidate the alteration of visfatin levels after periodontal treatments.

**Materials and methods:**

The database searched included Pubmed, Embase, Web of Science, and Cochrane Library. According to the Eligibility criteria, the records were screened and the eligible studies were included. The methodological qualities of the included case-controlled studies were assessed according to the Newcastle–Ottawa scale (NOS). The Methodological Index for Nonrandomized Studies (MINORS) was applied for assessing the qualities of the included clinical trials. The statistical analyses were processed using STATA 15.0.

**Results:**

Twenty-three studies were included in the statistical analyses. The meta-analysis showed significantly elevated visfatin levels of GCF, serum, and saliva in the periodontitis population compared with the controls (GCF: SMD = 5.201, 95% CI: 3.886–6.516, Z = 7.75, *P* < 0.05; Serum: SMD = 7.417, 95% CI: 3.068–11.767, Z = 3.34, *P* = *P* < 0.05; Saliva: SMD = 2.683, 95% CI: 1.202–4.163, Z = 3.34, *P* < 0.05). Visfatin levels of saliva serum and GCF were significantly decreased after periodontal treatment.

(Saliva: SMD = -1.338, 95% CI: -2.289—0.487, Z = 39.77, *P* < 0.05; Serum: SMD = -2.890, 95% CI: -5.300–0.480, Z = 2.35, *P* < 0.05; GCF: SMD = -6.075, 95% CI: -11.032—1.117, Z = 2.40, *P* = 0.016; I 2 = 95.9%, *P* < 0.05).

**Conclusions:**

Periodontitis elevated the visfatin levels in GCF, serum, and saliva. Additionally, GCF, serum, and saliva visfatin levels could be reduced after periodontal treatment.

## Introduction

Periodontitis is a common inflammatory disease induced by polymicrobial factors. The consequences of periodontal disease are frequently presented as gingival infection, alveolar bone destruction, as well as periodontal attachment loss [1]. The status of periodontitis strongly affected the quality of life. The destructed periodontal tissues not only give an annoying smell but also leads to aesthetic compromise, which influenced social life [2]. Additionally, the masticatory function declined, which brought difficulties in nutrition intake. Moreover, periodontitis has been reported to be associated with multiple systemic diseases, such as diabetes, cardiovascular disease, and cancer [3, 4]. The etiology of periodontitis has been extensively studied. Although the inflammation of periodontal tissue is supposed to be initialed by bacteria, the disproportionate and unbalanced host response to the microbial film and the inability of the host to resolve the inflammatory process is considered to be responsible for the occurred periodontal damage [5].

Adipokines are a complex cohort of cytokines produced by adipocytes, including leptin, adiponectin, resistin, and visfatin. etc. [6] It has been widely demonstrated that adipokines are involved in multiple internal events, such as energy metabolism, wound healing, as well as inflammatory reactions [7]. Visfatin, also identified as the pre-B-cell colony-enhancing factor (PBEF), is a 52-kDa protein promoting pre–B cell colony release from lymphocytes and improving the maturation of B lymphocytes [8]. As a pleiotropic cytokine, visfatin has roles in many processes, including insulin mechanisms, apoptosis, and inflammation regulation [9]. The association between visfatin and the pathogenesis of several systemic diseases, such as type 2 diabetes mellitus, polycystic ovary syndrome, and cardiovascular diseases has also been reported [10]. Additionally, in the process of inflammation, visfatin induces pro-inflammatory cytokines including tumor necrosis factor-alpha (TNF-α), interleukin (IL)- 1,6, and 8 [11], which played a critical role in the initiation and development of systemic inflammation from neutrophils, lymphocytes, and macrophages.

The relationship between adipokines and periodontitis has been reported. Previously, the present author published a meta-analysis regarding the association between periodontitis and serum levels of leptin and adiponectin, finding that elevated serum leptin and decreased serum adiponectin were related to periodontitis [12]. Recent year studies have shown that visfatin also played roles in the development of periodontitis. Multiple studies have been published to discover the possible links between visfatin and periodontitis, However, an evidence-based summative study was needed to provide a more precise evaluation. The present study gathered individual study results into a quantitative estimation, the aim was to compare the levels of visfatin in body fluids including serum, saliva, and gingival crevicular fluid (GCF) between periodontitis patients and healthy individuals, and to elucidate the alteration of visfatin levels after periodontal treatments.

## Methods

We conducted the present meta-analysis following the Preferred reporting items for systematic reviews and meta-analyses: the PRISMA statement [13]. The protocol of the present study was registered in the International platform of registered systematic review and meta-analysis protocols (INPLASY), the registration number is INPLASY202380125.

### Literature search

The database searched included Pubmed, Embase, Web of Science, and Cochrane Library, up to December 2022. The details of search strategies are presented in Table 1.

**Table 1:** The details of search strategies

**Pubmed**	("periodontal"[Title/Abstract] OR "periodontitis"[Title/Abstract]) AND ("visfatin"[Title/Abstract] OR "nicotinamide phosphoribosyltransferase"[Title/Abstract] OR "NAMPT"[Title/Abstract] OR "pre b cell colony enhancing factor"[Title/Abstract] OR "PBEF"[Title/Abstract])
**Embase**	(visfatin:ab,ti OR 'nicotinamide phosphoribosyltransferase':ab,ti OR nampt:ab,ti OR 'pre b cell colony enhancing factor':ab,ti OR pbef:ab,ti) AND (periodontitis:ab,ti OR periodontal:ab,ti)
**Web of science**	(TS = (periodontal) OR TS = (periodontitis)) AND (TS = (Visfatin) OR TS = (nicotinamide phosphoribosyltransferase) OR TS = (NAMPT) OR TS = (pre b cell colony enhancing factor) OR TS = (PBEF))
**Cochrane Library**	((periodontal):ti,ab,kw OR (periodontal disease):ti,ab,kw (Word variations have been searched))AND ((visfatin):ti,ab,kw OR (nicotinamide phosphoribosyltransferase):ti,ab,kw OR (NAMPT):ti,ab,kw OR (pre b cell colony enhancing factor):ti,ab,kw OR (PBEF):ti,ab,kw (Word variations have been searched))

### Eligibility criteria

The inclusion criteria for the included studies were as follows: (1) the observational studies comparing visfatin levels in serum, saliva, or GCF between periodontitis patients and periodontally healthy individuals; (2) Clinical trials comparing visfatin levels in serum, saliva, or GCF before and after periodontal treatments; (3) Studies with sufficient data for the statistical analyses; and (4) Studies in English or Chinese.

The exclusion criteria were as follows: (1) Conference abstracts; (2) Trial registry records; and (3) those with repeated data.

### Records screen

Search results were downloaded to EndNote. According to the Eligibility criteria, the records were collated and separately reviewed by two independent authors. A third author was consulted when consensus on appropriateness was debated. The titles and abstracts were screened first and the full-text paper screen was conducted next.

### Data extraction

The information on study characteristics and outcomes was extracted, including (1) Name of the first author and year of publication; (2)Country; (3) study design; (4) Group and size; (5)Age; (6) Body mass index (BMI); (7) Systemic conditions; (8)Type of periodontal treatments; (9) The time interval after periodontal treatment; and (10) Serum, saliva, or GCF levels of visfatin.

### Quality assessment

The methodological qualities of the included case-controlled studies were assessed according to the Newcastle–Ottawa scale (NOS). Three dimensions including selection, comparability, and exposure were considered. In the section on comparability, the most important factor was identified to be BMI. Eight items were graded, with a score range of 0–9 points. Overall, studies with final scores of 1–3, 4–6, and 7–9 were considered to be of low, moderate, and high qualities [14]. Additionally, the Methodological Index for Nonrandomized Studies (MINORS) was applied for assessing the qualities of the included clinical trials. A total of 12 items were considered, and 0–2 points could be graded for each item. As in the present study we only evaluated the quality of single-arm designs, the first 8 items were employed. The final scores ranged from 0 to 16 points. Studies with scores 0–5, 6–10, and 11–16 were considered to be of low, moderate, and high qualities [12].

### Meta-analysis

The data of visfatin levels were presented as mean (M) ± standard deviation (SD). The results in the form of median (minimum – maximum) were shifted to M ± SD according to the estimation method reported by Hozo et al. [15]. The standard mean difference (SMD) and corresponding 95% confidence interval (CI) were calculated. Heterogeneity was estimated by chi-square and I^2^. Random-effects model was used when the P value < 0.05 and I^2^ > 50%. Sensitivity analyses were performed to test the robustness. The statistical analyses were processed using STATA 15.0.

## Results

### Characteristics and quality assessment

**Fig. 1:**
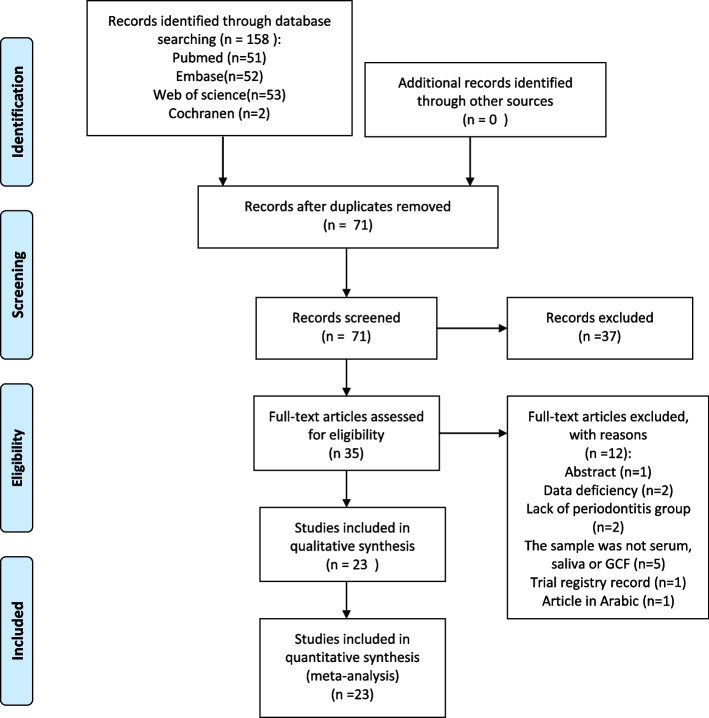
The flow diagram of the selection process

The flow diagram of the selection process was presented in Fig. 1. Finally, 23 studies were gained according to the eligibility criteria, including 14 case-controlled studies, 3 clinical trials, and 6 studies that employed both clinical trials and case-controlled designs. The origin of the studies included Iran, Saudi Arabia, Turkey, China, India, Iraq, and the USA. The descriptive information characteristic was presented in Table 2. 20 case-controlled studies were evaluated through NOS. The final scores ranged from 4 to 8 (Table 3). 17 studies were considered to be of moderate quality, and 3 studies were of high quality. MINORS was employed for the 9 clinical trials in the present study. final scores ranged from 11 to 15, indicating all of the 9 clinical trials were of high quality (Table 4).

**Table 2:** Characteristics of the included studies

Author	Year	Country	Study design	Group and size	Age	BMI	Systematic conditions	Periodontal treatment	Time interval	Sample
Abolfazli, N [16]	2015	Iran	Single-armed clinical trial	Periodontitis (18)	41.16 ± 7.59	24.35 ± 0.63	Healthy	SRP	4 weeks	Serum and saliva
Bahammam, M. A [17]	2018	Saudi Arabia	Case-controlled	Control(20)/Periodontitis and T2DM (20)/Periodontitis(20)/Periodontitis and T2DM using simvastatin(20)	32–50	NA	Healthy/T2dM/Healthy/T2dM	NA	NA	GCF
Çetiner, D [18]	2019	Turkey	Case-controlled	Periodontitis (9)/Control(10)/Obese and periodontitis (21)/Obese control (10)	44.67 ± 10.87/46.50 ± 12.0 ( Non-obese); 44.22 ± 6.7/27.80 ± 3.12(Obese)	BMI < 25 ( Non-obese); BMI ≥ 30(Obese)	Healthy/Healthy/Obese/Obese	NA	NA	GCF
Chen, F. [19]	2018	China	Case-controlled	Periodontitis (79)/Control (25)	Male (Female): 30(27) /30(25)	NA	Healthy/Healthy	NA	NA	Serum
Coutinho, A [20]	2021	India	Case-controlled	Periodontitis (20)/Control (20)	20–50	18.5–29.9	Healthy/Healthy	NA	NA	Saliva
Hamdi, A. Q [21]	2021	Iraq	Case-controlled	Periodontitis and T2DM (41)/Control (49)	60 ± 14/61.8 ± 12.5	NA	T2DM/Healthy	NA	NA	Serum
Mishra, V [22]	2016	India	Case-controlled; Clinical trial	Control(14)/Periodontitis (14)/Periodontitis and T2DM (14)	32.43/41.7/48.07	NA	Healthy/Healthy/T2DM	SRP	4 weeks	GCF
Mohamed, H. G [23]	2015	USA	Case-controlled	T2DM and periodontitis (54)/T2DM control(24)/ Periodontitis (30) Control (44)	54.76 ± 1.38/50.79 ± 2.09/55.37 ± 1.77/47.15 ± 1.56	NA	Healthy/Healthy/T2DM/T2DM	NA	NA	GCF
Mopidevi, A [24]	2019	India	Case-controlled; Single-armed clinical trial	Periodontitis (10)/Control (10)	43.9 ± 9.65/44.1 ± 8.41	24.68 ± 2.65/24.37 ± 2.54	Healthy/Healthy	SRP and Surgical periodontal therapy	12 weeks	Saliva
Özcan, E.(A) [25]	2016	Turkey	Case-controlled; Single-armed clinical trial	Periodontitis (17)/Control (15)	41.52 ± 6.34 /44.06 ± 5.36	24.12 ± 0.78/ 23.62 ± 1.53	Healthy/Healthy	SRP	3 months and 6 months	Saliva
Özcan, E.(B) [26]	2016	Turkey	Case-controlled	Periodontitis (27)/Control (18)	41.59 ± 7.23/37.72 ± 9.02	24.74 ± 1.22/24 ± 1.04	Healthy/Healthy	NA	NA	GCF
Özcan, E [27]	2015	Turkey	Case-controlled	Periodontitis (25)/Control (23)	32.56 ± 7.92/34.50 ± 7.09	23.50 (21.20–26.15)/24.55(21.92–28.17)	Healthy/Healthy	NA	NA	Saliva
Paul, R [28]	2020	India	Case-controlled	Periodontitis (30)/Control (30)	30–55	NA	Healthy/Healthy	NA	NA	GCF
Pradeep, A. R [29]	2011	India	Case-controlled	Periodontitis (15)/Control (10)/Gingivitis(15)	32.6 ± 7.5 /28.70 ± 3.6/ 28.5 ± 3.1	NA	Healthy/Healthy	NA	NA	Serum and GCF
Pradeep, A. R [30]	2012	India	Case-controlled	Control(10/Periodontitis and T2DM(10)/Periodontitis (10)	31.33 ± 3.478/38.80 ± 5.634/32.60 ± 7.59	27.28 ± 2.41 /28.46 ± 2.72 /28.59 ± 3.14	Healthy/T2DM/Healthy	NA	NA	Serum and GCF
Raghavendra, N. M [31]	2012	India	Case-controlled; Single-armed clinical trial	Periodontitis (15)/Control (15)	38.80 ± 5.634/31.33 ± 3.478	NA	Healthy/Healthy	SRP	8 weeks	Serum and GCF
Rezaei, M [32]	2019	Iran	Case-controlled	SLE and periodontitis (15)/SLE control(15)/ Periodontitis (15) Control (15)	44.17 ± 3.21/44.89 ± 3.07/45.03 ± 3.14/44.99 ± 3.26	21.89 ± 1.83/22.02 ± 1.17/23.05 ± 1.65/22.65 ± 2.10	SLE/SLE/Healthy/Healthy	NA	NA	GCF
Saljoughi, F [33]	2020	Iran	Case-controlled	PCOS and periodontitis (30)/PCOS control(25)/ Periodontitis (23) Control (32)	45.2 ± 3.2/45.3 ± 3.0/45.3 ± 3.1/45.5 ± 3.3	22.43 ± 1.79/22.13 ± 1.58/22.38 ± 1.71/22.07 ± 1.50	PCOS/PCOS/Healthy/Healthy	NA	NA	GCF
Saseendran, G [34]	2021	India	Case-controlled; Clinical trial	Control (16)/Gingivitis(16)/Periodontitis(16)	25–50	NA	Healthy/Healthy/Healthy	SRP	2 months	Saliva
Tabari, Z. A [35]	2014	Iran	Case-controlled	Periodontitis (20)/Control (20)	38.45 ± 9.98/33.85 ± 6.84	23.66 ± 2.99/21.88 ± 2.56	Healthy/Healthy	NA	NA	Saliva
Tabari, Z. A [36]	2015	Iran	Case-controlled; Single-armed clinical trial	Periodontitis (20)/Control (20)	38.45 ± 9.98/33.85 ± 6.84	23.66 ± 2.99/21.88 ± 2.56	Healthy/Healthy	SRP	1 months	Saliva
Wu, Y [37]	2015	China	Randomized clinical trial	T2DM and periodontitis (23)/T2DM and periodontitis (23)	54.09 ± 6.57/55.52 ± 5.22	22.22 ± 0.64/22.14 ± 0.72	T2DM/T2DM	SRP	3 months and 6 months	Serum and GCF
Ziaei, N [38]	2020	Iran	Single-armed clinical trial	T2DM and periodontitis (20)	51.85 ± 6.42	26.8 ± 1.5	T2DM	SRP	3 months	Saliva

**Table 3:** Quality assessment for case-controlled studies

Author and year	Selection	Comparability	Exposure	Total
Bahammam, M. A 2018 [17]	●●○●	○●	●●○	7/9
Çetiner, D. 2019 [18]	●●○●	●●	●●○	8/9
Chen, F. 2018 [19]	●○●●	○●	●●○	7/9
Coutinho, A 2021 [20]	●○○●	●●	●●○	6/9
Hamdi, A. Q. 2021 [21]	●○○●	○○	●●○	4/9
Mishra, V. 2016 [22]	●○○●	○●	●●○	5/9
Mohamed, H. G. 2015 [23]	●○○●	○●	●●○	5/9
Mopidevi, A. 2019 [24]	●○○●	●●	●●○	6/9
Özcan, E (A). 2016 [25]	●○○●	●●	●●○	6/9
Özcan, E.(B). 2016 [26]	●○○●	●●	●●○	6/9
Özcan, E. 2015 [27]	●○○●	●●	●●○	6/9
Paul, R 2020 [28]	●○○●	○●	●●○	5/9
Pradeep, A. R. 2011 [29]	●○○●	○●	●●○	5/9
Pradeep, A. R. 2012 [30]	●○○●	●●	●●○	6/9
Raghavendra, N. M. 2012 [31]	●○○●	○●	●●○	5/9
Rezaei, M. 2019 [32]	●○○●	●●	●●○	6/9
Saljoughi, F. 2020 [33]	●○○●	●●	●●○	6/9
Saseendran, G. 2021 [34]	●○○●	○●	●●○	5/9
Tabari, Z. A. 2014 [35]	●○○●	●●	●●○	6/9
Tabari, Z. A. 2015 [36]	●○○●	●●	●●○	6/9

● = the item was fulfilled; ○ = the item was not fulfilled

**Table 4:** Quality assessment for clinical trials

Author and year	1	2	3	4	5	6	7	8	Total
Abolfazli, N 2015 [16]	●	◐	●	●	●	◐	●	○	12/16
Mishra, V. 2016 [22]	●	◐	●	●	●	◐	●	●	14/16
Mopidevi, A. 2019 [24]	●	○	●	●	●	●	●	◐	13/16
Özcan, E.(A) 2016 [25]	●	◐	●	●	●	●	●	○	13/16
Raghavendra, N. M. 2012 [31]	●	○	●	●	●	◐	●	○	11/16
Saseendran, G2021 [34]	●	◐	●	●	●	◐	●	○	14/16
Tabari, Z. A. 2015 [36]	●	◐	●	●	●	◐	●	○	14/16
Wu, Y. 2015 [37]	●	◐	●	●	●	●	●	○	15/16
Ziaei, N. 2020 [38]	●	○	●	●	●	●	●	○	14/16

● = the item was reported and adequate; ○ = the item was not reported; ◐ = the item was reported but inadequate

### Meta-analysis

**Fig. 2:**
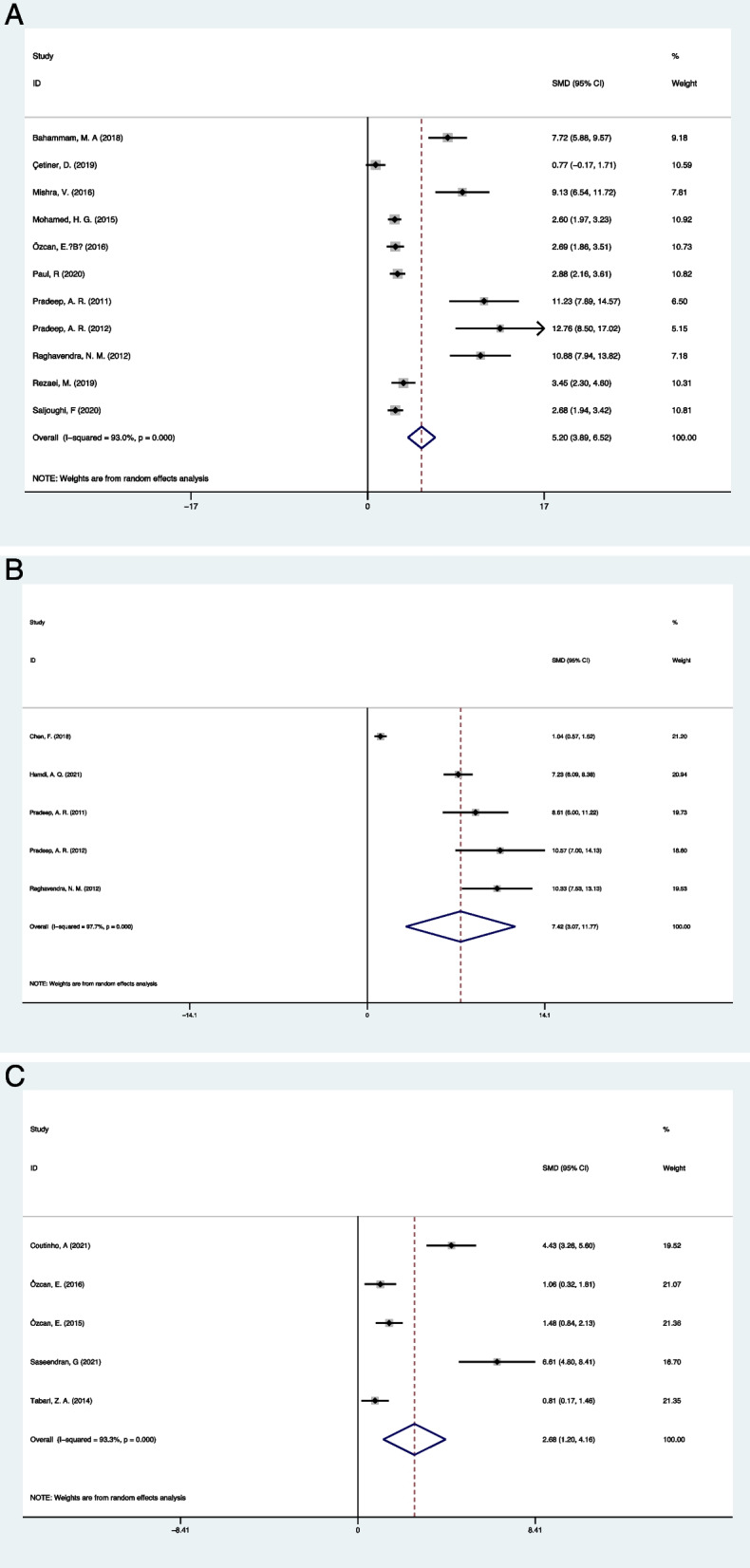
GCF, serum, and saliva visfatin levels between periodontitis patients and healthy individuals. **A** GCF; **B** Serum; **C** Saliva

Eleven studies reported the comparison of visfatin levels in GCF between periodontitis patients and healthy individuals. The meta-analysis showed significantly increased GCF visfatin levels in the periodontitis population (SMD = 5.201, 95% CI: 3.886–6.516, Z = 7.75, *P* < 0.05; I ^*2*^ = 93.0%, *P* < 0.05 Fig. 2A). 5 studies reported the comparison of visfatin levels in serum between periodontitis patients and healthy individuals. Also, the meta-analysis showed significantly increased serum visfatin levels in the periodontitis population (SMD = 7.417, 95% CI: 3.068–11.767, Z = 3.34, *P* = 0.001; I ^*2*^ = 97.7%, *P* < 0.05 Fig. 2B). We found the study conducted by Chen, F et al. [19] contributed to the heterogeneity. When the study was removed, the heterogeneity was significantly decreased, and the result was not influenced (SMD = 8.005, 95% CI: 7.058–8.953, Z = 16.56, *P* < 0.05; I ^*2*^ = 54.4%, *P* = 0.087). 5 studies reported the comparison of visfatin levels in saliva between periodontitis patients and healthy individuals. The meta-analysis showed significantly increased saliva visfatin levels in the periodontitis population (SMD = 2.683, 95% CI: 1.202–4.163, Z = 3.34, *P* = 0.001; I ^*2*^ = 93.3%, *P* < 0.05 Fig. 2C).

**Fig. 3:**
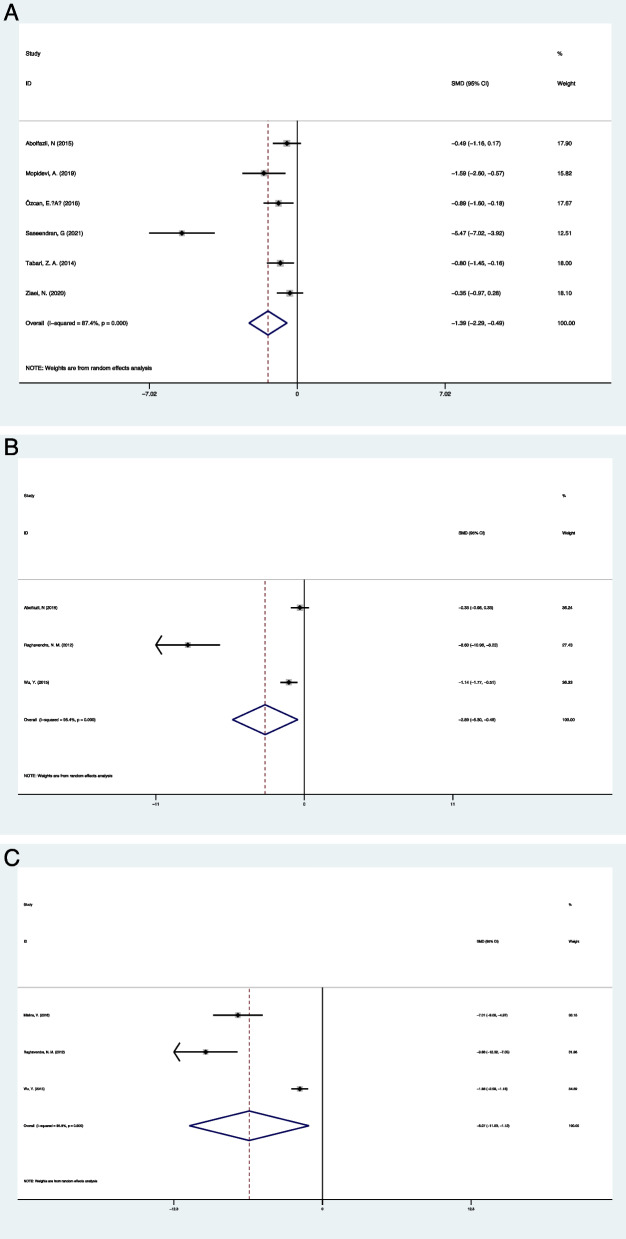
The change of visfatin levels after periodontal treatment. **A** Saliva; **B** Serum; **C** GCF

Six clinical trials reported the change in saliva visfatin levels after periodontal treatments. The results of the meta-analysis showed a significantly declined saliva visfatin levels after periodontal treatment compared with the baseline (SMD = -1.338, 95% CI: -2.289—0.487, Z = 39.77, *P* = 0.003; I ^*2*^ = 87.4%, *P* < 0.05 Fig. 3A). the study conducted by Saseendran, G et al. [34] contributed to the heterogeneity. When the study was removed, the heterogeneity was significantly decreased, and the result was not influenced (SMD = -0.710, 95% CI: -1.023—0.397, Z = 16.56, *P* < 0.05; I ^*2*^ = 17.8%, *P* = 0.301). 3 studies for each described the change of visfatin levels in serum and GCF after periodontal treatment. The results of the meta-analysis also showed significantly declined serum and GCF visfatin levels after periodontal treatment compared with the baseline (Serum: SMD = -2.890, 95% CI: -5.300–0.480, Z = 2.35, *P* = 0.0019; I 2 = 95.4%, *P* < 0.05 Fig. 3B; GCF: SMD = -6.075, 95% CI: -11.032—1.117, Z = 2.40, *P* = 0.016; I 2 = 95.9%, *P* < 0.05 Fig. 3C).

**Fig. 4:**
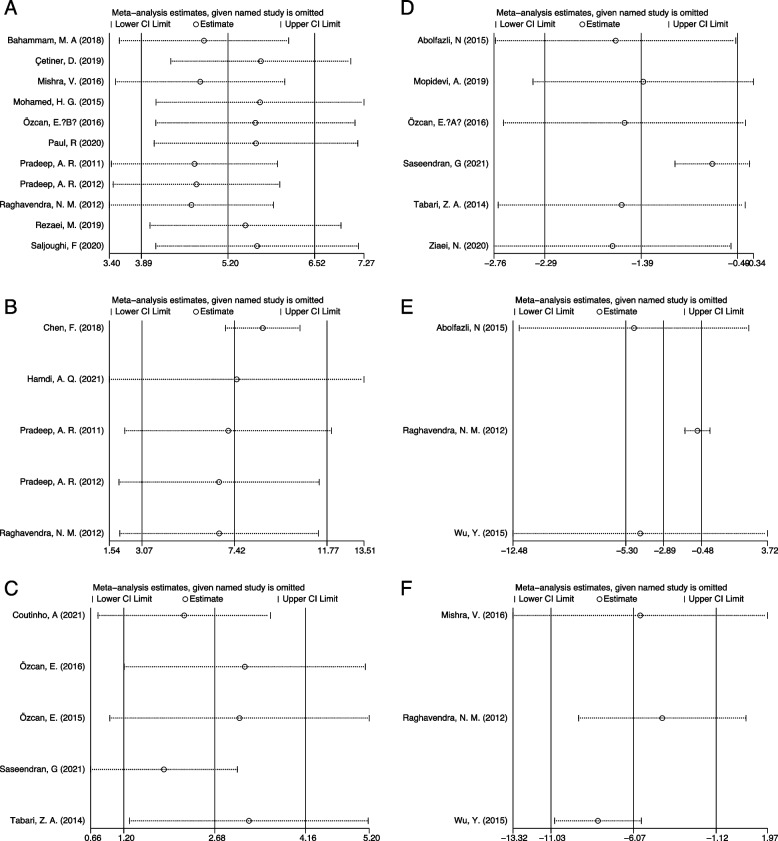
Sensitive analysis. **A** GCF visfatin; **B** Serum visfatin; **C** Saliva visfatin; **D** The change of saliva visfatin after periodontal treatment; **E** The change of serum visfatin after periodontal treatment; **F** The change of GCF visfatin after periodontal treatment

The sensitivity analyses were proceeded by removing a single study each time and observing the influence of each study on the result. The results of sensitivity analyses were presented in Fig. 4, according to which the robustness of the results was confirmed.

### Publication bias

Egger's linear regression test was used to examine the potential publication bias. We found a significant publication bias in the comparison of visfatin levels in GCF between periodontitis patients and healthy individuals (*P* < 0.05). For other comparisons, Egger’s linear regression test was not conducted because the number of included studies was limited (n < 10).

## Discussion

Increasing evidence in the literature indicated the associations between adipocytokines and periodontitis. In 2016, Zohaib Akram et al. published the first meta-analysis researching the cytokine profile in periodontitis patients with and without Obesity, finding increased resistin levels in obese individuals with periodontitis than nonobese periodontitis subjects [39]. Another evidence-based study conducted by the team found that periodontitis patients were presented with elevated levels of GCF or serum resistin, indicating resistin might be used as a potential biomarker for chronic periodontitis [40]. In 2017, the author of the present study published a systematic review and meta-analysis regarding the circulating leptin and adiponectin levels in the periodontitis population, the results showed elevated serum levels of leptin and decreased serum levels of adiponectin were found in periodontitis patients, and periodontal treatment influenced the circulating leptin and adiponectin [12].

The present study is the first meta-analysis concentrated on the association between visfatin and periodontitis. 23 studies were examined in the present study, and the results showed that visfatin levels in GCF, serum, and saliva were significantly elevated in the periodontitis population. Additionally, we also found that GCF, serum, and saliva visfatin levels could be reduced after periodontal treatment.

Human visfatin was first discovered in 1994 as a novel gene isolated from a human peripheral blood lymphocyte cDNA library [41]. Visfatin has been reported as the pleiotropic mediator that exerts metabolic and immune functions [42]. Subsequent work has shown visfatin to be of potent destructive and proinflammatory properties. The protein played a key role in the persistence of inflammation through the inhibition of apoptosis and neutrophils [43]. A variety of inflammatory processes and systematic disorders have been associated with the upregulated expression of visfatin, such as sepsis [44], inflammatory bowel diseases [45], rheumatoid arthritis [46], diabetes [47], as well as cardiovascular diseases [48].

Periodontitis is a common bacterial infection associated with multiple risk factors. Despite the microorganisms' roles as etiologic agents, systemic conditions and inflammatory situations have also contributed to the prevalence and severity of periodontitis [49]. Periodontal supporting tissues were destroyed in periodontitis through the main periodontal pathogens Porphyromonas gingivalis (Pg), Fusobacterium nucleatum (Fn), and the toxic products. On the other hand, the systemic imbalance of pro-inflammatory/anti-inflammatory immune regulation also plays an important role in bone tissue destruction. Studies have shown that visfatin can affect bone metabolism and is a risk factor for osteoporosis [50]. In the present study, we found elevated visfatin levels in body fluids in periodontitis patients, indicating that visfatin might be involved in the inflammatory responses of periodontitis.

Among the 3 kinds of physiological fluids investigated in the present study, GCF was mostly reported. GCF is the fluid and the inflammatory exudate secreted from the blood vessels in the gingival corium, subjacent to the epithelium lining of the dentogingival space. Cytokines presented in GCF have been proposed as potential diagnostic or prognostic markers of periodontal destruction [51]. The present study found elevated visfatin in GCF in periodontitis. The increased visfatin in CGF could be secreted by the inflamed periodontal ligament cells or the activated Porphyromonas gingivalis [28]. In sequence, the locally secreted high-level visfatin entered the peripheral blood circulation through the epithelium of the periodontal pocket, the proliferating capillaries, as well as the loose connective tissue. In comparison to GCF, saliva can be collected in an easy, non‐invasive, time-saving manner with minimally trained personnel [52]. Salivary proteomics has been a growing topic of research due to its characteristic to be a diagnostic tool for both oral and systemic diseases. And GCF is considered to be a better source for assaying specific dental or periodontal biomarkers of oral conditions due to its ability to be site specific [53]. Additionally, the present study also suggested the upregulated serum visfatin levels in the population with periodontitis, indicating that periodontal inflammation may modulate the systemic levels of visfatin. On the one hand, the increased circulating visfatin could be induced by the locally secreted high-leveled GCF and saliva visfatin. Additionally, another possible explanation for the finding is that peripheral B cells, T cells, monocytes, macrophages, and neutrophils, rather than adipocytes, release visfatin in the body because systematic inflammation could be activated by periodontitis [54].

The limitations of the presented study include the lack of enough included studies, especially randomized controlled studies. Although 9 clinical trials were included, only 3–6 studies were evaluated in each comparison for the change of visfatin levels after periodontal treatment. And, only single-armed results were applied in the meta-analysis to ensure consistency. Besides, significant heterogeneities were observed, and in some comparisons, we failed to find the exact source of heterogeneity, therefore the results should be interpreted with caution. Moreover, although the present study o included studies with case-controlled designs, the results of each study were considered cross-sectional, therefore the cause-effect relationship between visfatin and periodontitis could not be perfectly elucidated.

In conclusion, the present study supports the findings that periodontitis elevated the visfatin levels in GCF, serum, and saliva. Additionally, GCF, serum, and saliva visfatin levels could be reduced after periodontal treatment. Further large-scale homogeneous studies were still needed to prove if visfatin could be used as a potential biomarker to predict periodontitis.

## Data Availability

The datasets used and/or analyzed during the current study available from the corresponding author on reasonable request.
